# D4F alleviates macrophage-derived foam cell apoptosis by inhibiting the NF-κB-dependent Fas/FasL pathway

**DOI:** 10.1038/s41598-017-07656-0

**Published:** 2017-08-04

**Authors:** Hua Tian, Shu-tong Yao, Na-na Yang, Jie Ren, Peng Jiao, Xiangjian Zhang, Dong-xuan Li, Gong-an Zhang, Zhen-fang Xia, Shu-cun Qin

**Affiliations:** 1 Key Laboratory of Atherosclerosis in Universities of Shandong, Institute of Atherosclerosis, Taishan Medical University, Taian, 271000 China; 2 College of Basic Medical Sciences, Taishan Medical University, Taian, 271000 China; 3 Institute of Cardiovascular Disease, General Hospital of Jinan Military Region, Jinan, 250022 China; 4 Hebei Collaborative Innovation Center for Cardio-cerebrovascular Disease and Hebei Key Laboratory of Vascular Homeostasis, Shijiazhuang, 050000 China

## Abstract

This study was designed to explore the protective effect of D4F, an apolipoprotein A-I mimetic peptide, on nuclear factor-κB (NF-κB)-dependent Fas/Fas ligand (FasL) pathway-mediated apoptosis in macrophages induced by oxidized low-density lipoprotein (ox-LDL). Our results showed that ox-LDL induced apoptosis, NF-κB P65 nuclear translocation and the upregulation of Fas/FasL pathway-related proteins, including Fas, FasL, Fas-associated death domain proteins (FADD), caspase-8 and caspase-3 in RAW264.7 macrophages, whereas silencing of Fas blocked ox-LDL-induced macrophage apoptosis. Furthermore, silencing of P65 attenuated macrophage apoptosis and the upregulation of Fas caused by ox-LDL, whereas P65 expression was not significantly affected by treatment with Fas siRNA. D4F attenuated the reduction of cell viability and the increase in lactate dehydrogenase leakage and apoptosis. Additionally, D4F inhibited ox-LDL-induced P65 nuclear translocation and upregulation of Fas/FasL pathway-related proteins in RAW264.7 cells and in atherosclerotic lesions of apoE^−/−^ mice. However, Jo2, a Fas-activating monoclonal antibody, reversed the inhibitory effect of D4F on ox-LDL-induced cell apoptosis and upregulation of Fas, FasL and FADD. These data indicate that NF-κB mediates Fas/FasL pathway activation and apoptosis in macrophages induced by ox-LDL and that D4F protects macrophages from ox-LDL-induced apoptosis by suppressing the activation of NF-κB and the Fas/FasL pathway.

## Introduction

Atherosclerosis (AS) is a chronic inflammatory disease of the arterial wall. Macrophages ingest an excess amount of oxidized low-density lipoprotein (ox-LDL) and are converted into foam cells, which are the characteristic components of atherosclerotic plaques and are closely associated with the development and progression of AS^1^. Evidence has demonstrated that macrophage apoptosis reduces lesion size in early atherosclerotic lesions^2, 3^, whereas apoptosis of macrophage-derived foam cells in advanced lesions promotes lipid core enlargement and leads to inflammation, necrosis and even plaque rupture, which is the main cause of acute coronary syndromes, such as unstable angina, acute myocardial infarction and sudden cardiac death^4–6^. Thus, inhibition of macrophage apoptosis may be an effective therapeutic strategy against plaque rupture.

The death receptor family apoptotic pathway plays a key role in apoptosis^7^, and the Fas receptor (Fas)/Fas ligand (FasL) pathway is important and widely recognized in this process^8^. Fas (CD95), a 45 kDa transmembrane protein, belongs to the death receptor (DR) family, which is a subset of the tumor necrotic factor receptor superfamily. FasL binds to Fas as a trimer at the cell surface and induces the recruitment of Fas-associated death domain proteins (FADD, an adaptor protein of Fas) via interaction with the death domain (DD) on both proteins. FADD also has a death effector domain (DED) that facilitates its interaction with other DED-containing proteins, such as caspase-8/10^9^. Once bound to FADD, caspase-8/10 become activated and in turn activate the downstream effector caspase-3 to form the death-inducing signaling complex (DISC), which then triggers cell apoptosis^10, 11^. The apoptotic cells in carotid plaques express Fas and FasL^12^, and Fas transduced with adenovirus can reduce the number of cells in the fibrous cap and increase thrombus rupture and bleeding inside the plaque, thereby increasing plaque vulnerability^13^. Additionally, it has been reported that ox-LDL-induced macrophage apoptosis is mediated by the Fas/FasL death receptor signaling pathway and may be blocked by antagonistic Fas antibody^14^. These data indicate that Fas/FasL pathway-mediated apoptosis and the development of AS are closely related.

D4F is an 18-amino-acid mimetic peptide of apolipoprotein A-I (apoA-I), an important functional component of high-density lipoprotein (HDL). It does not share sequence homology with apoA-I, but it possesses a class A amphipathic helix that allows it to bind lipids similar to apoA-I^15, 16^. D4F has been demonstrated to have anti-atherogenic effects, such as improving reverse cholesterol transport (RCT) in macrophages from apoE^−/−^ mice^17^ and in RAW264.7 cells^18^, preventing the oxidation of low-density lipoprotein (LDL), decreasing ox-LDL-induced monocyte chemotactic activity and increasing the anti-inflammatory properties of HDL. Additionally, D4F has been confirmed to reduce atherosclerotic lesion formation in mice independent of plasma cholesterol, increase levels of pre-βHDL (the fraction that is most important in RCT)^19–23^ and significantly enhance endothelial progenitor cell proliferation, migration and adhesion to repair the injured endothelia^24^. Our recent work has also shown that D4F reduces ox-LDL-induced cytotoxicity of human umbilical vein endothelial cells (HUVECs) by preventing the downregulation of pigment epithelium-derived factor^25^, and alleviates macrophage-derived foam cell apoptosis by inhibiting CD36 expression and the endoplasmic reticulum stress-C/EBP homologous protein pathway^26^. In this research, we investigated the inhibitory effect of D4F on NF-κB activation and subsequent Fas/FasL death receptor pathway-mediated macrophage apoptosis.

## Results

### Ox-LDL induces apoptosis, P65 nuclear translocation and the activation of Fas/FasL pathway in RAW264.7 cells

**Figure 1:**
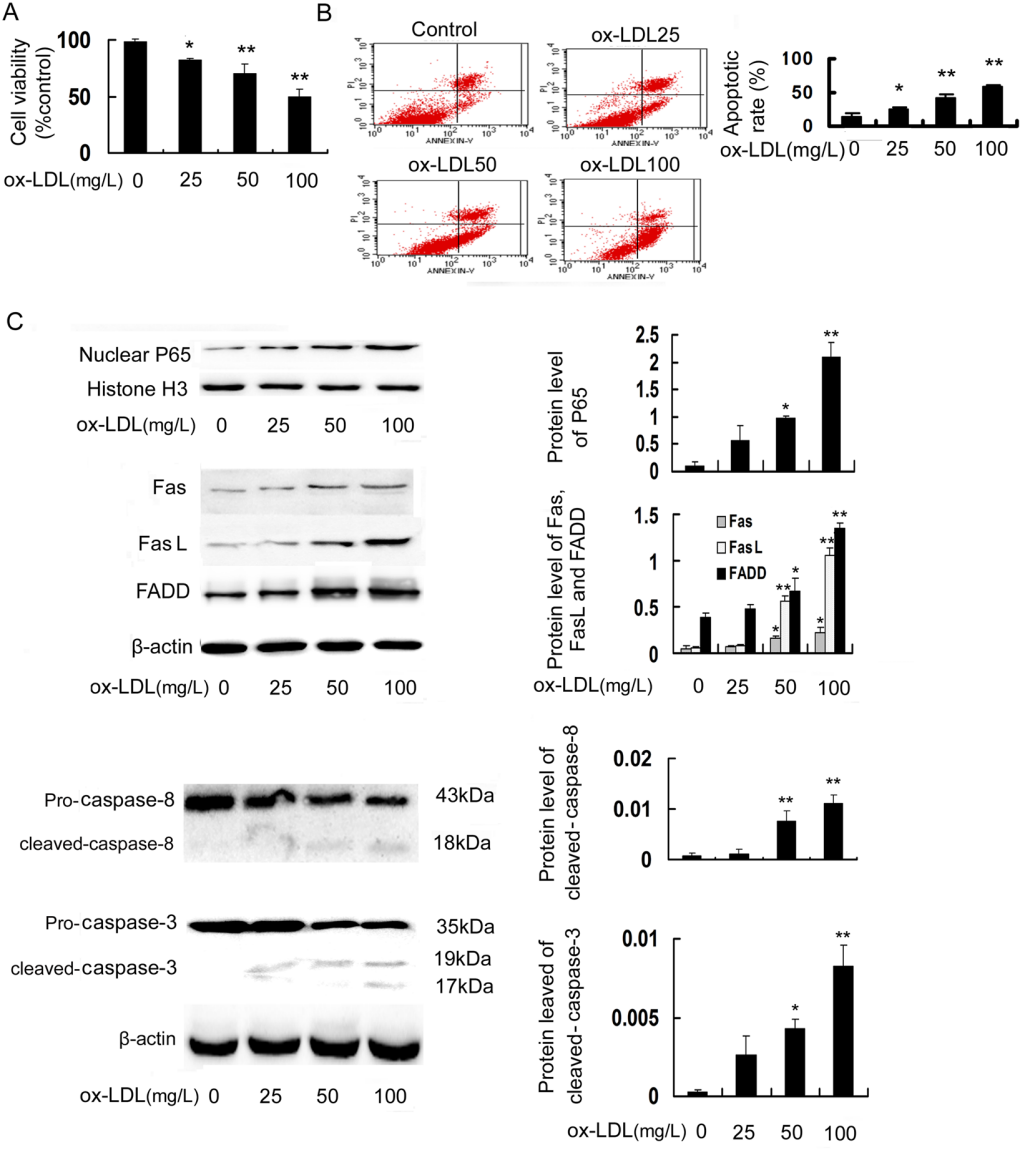
Ox-LDL induces apoptosis, P65 nuclear translocation and the activation of Fas/FasL pathway in RAW264.7 cells. Cells were incubated with ox-LDL (25, 50 and 100 mg/L) for 24 h. (**A**) Cell viability was measured using the MTT assay and expressed as a percentage of the control. (**B**) Cell apoptosis was detected using flow cytometry, and the total apoptotic cells (early and late-stage apoptosis) are presented on the right side of the panel (Annexin V staining alone or together with PI). (**C**) The protein levels of P65 in nuclear extracts and Fas/FasL pathway-related molecules were evaluated by Western blot. Data are expressed as the mean ± SD of at least three independent experiments. *P < 0.05 and **P < 0.01 versus vehicle-treated control.

Cell viability and apoptosis were assessed by the MTT assay and Annexin V-FITC/PI double-staining assay, respectively. As seen in Fig. 1A, treatment with ox-LDL at different concentrations (25, 50 and 100 mg/L) for 24 h decreased cell viability to 81.8%, 69.9% and 49.2%, respectively, compared with the control group. Additionally, the proportion of apoptotic cells was increased by ox-LDL in a dose-dependent manner (Fig. 1B). Additionally, oil red O staining and intracellular total cholesterol (TC) quantitative assay indicated that ox-LDL, but not LDL, remarkably induced lipid accumulation, which is an important inducer of macrophage apoptosis (Supplementary Fig. S1).

Subsequently, we detected the expression levels of P65 and Fas/FasL pathway-related molecules in ox-LDL-treated RAW264.7 cells by Western blot. As shown in Fig. 1C, the P65 levels in the nuclei and the protein levels of Fas, FasL and FADD as well as the active forms of caspase-8 and caspase-3 (cleaved-caspase-8 and cleaved-caspase-3) were increased in response to treatment with ox-LDL in a concentration-dependent manner.

### P65 mediates Fas upregulation and apoptosis in RAW264.7 cells induced by ox-LDL

**Figure 2:**
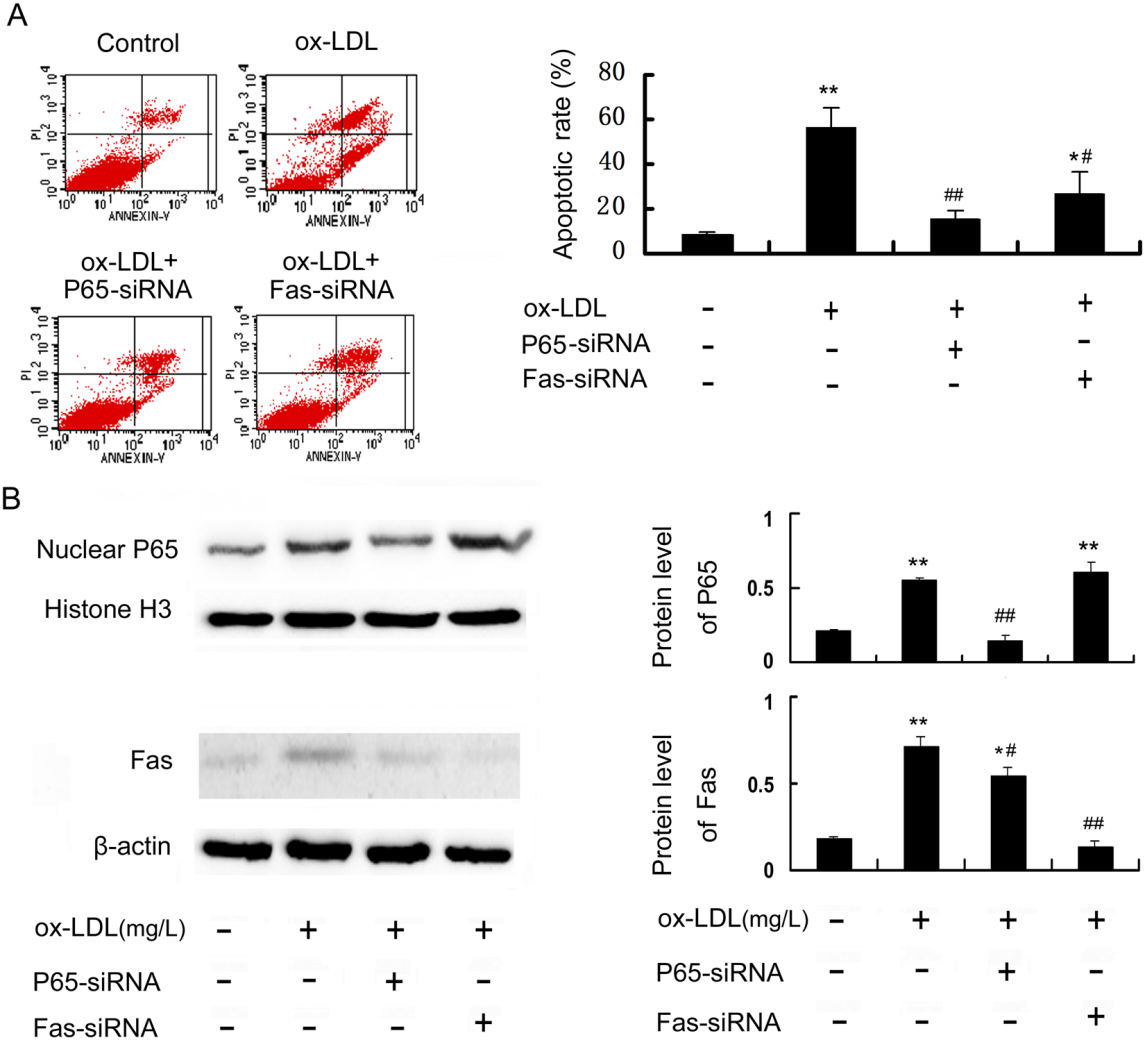
P65 mediates Fas upregulation and apoptosis in RAW264.7 cells induced by ox-LDL. Cells were transfected with siRNA against P65 or Fas, followed by treatment with 100 mg/L ox-LDL for 24 h. Cell apoptosis and the protein expression of P65 and Fas were detected using flow cytometry (**A**) and Western blot (**B**), respectively. Data are expressed as the mean ± SD of at least three independent experiments. *P < 0.05 and **P < 0.01 versus vehicle-treated control; ^#^ *P* < 0.05 and ^##^ *P* < 0.01 versus ox-LDL treatment.

Both Fas and FasL promoter regions contain NF-κB binding sites in NK cells, T lymphocytes, HepG2 cells, etc.^27–30^. However, little is known about the interrelationship between NF-κB and Fas in macrophage apoptosis. In the present study, to further clarify the role of P65 and Fas in macrophage apoptosis and their relationship, we silenced the expression of P65 and Fas by siRNA and then explored their inhibitory effects on cell apoptosis and their protein expression levels. The results showed that both P65 and Fas silencing efficiently reduced ox-LDL-induced apoptosis (Fig. 2A) and the corresponding protein expression (Fig. 2B). Interestingly, Fas protein expression was decreased by P65 siRNA, whereas P65 expression was not affected by Fas siRNA (Fig. 2B), indicating that P65 may mediate the activation of the Fas pathway, which then causes cell apoptosis.

### D4F attenuates apoptosis, P65 nuclear translocation and the activation of Fas/FasL pathway in RAW264.7 cells induced by ox-LDL

**Figure 3:**
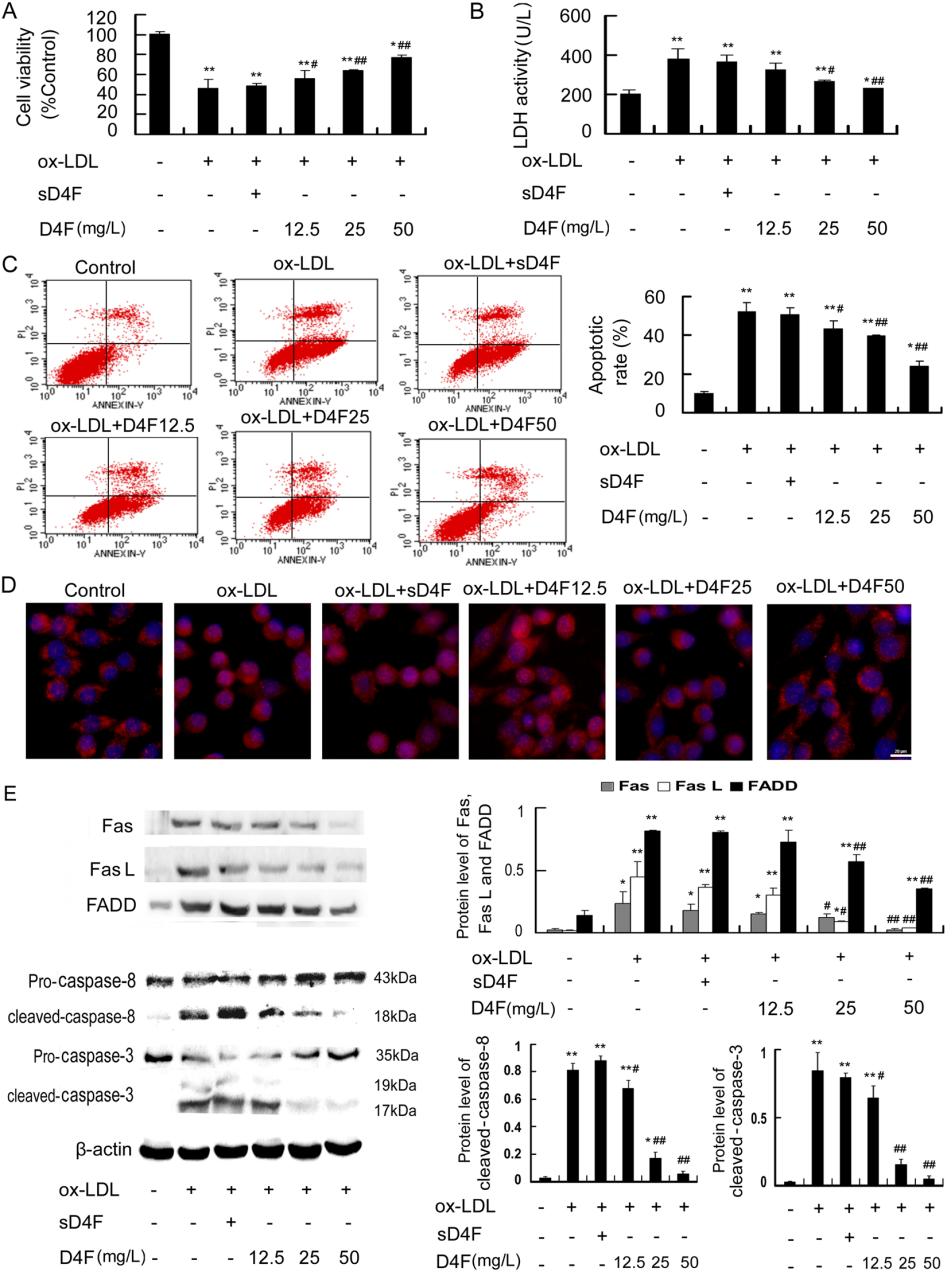
D4F attenuates apoptosis, P65 nuclear translocation and the activation of Fas/FasL pathway in RAW264.7 cells induced by ox-LDL. RAW264.7 cells were pretreated with D4F (12.5, 25 and 50 mg/L) or scrambled D4F (sD4F, 50 mg/L), the inactive control peptide, for 1 h followed by incubation with ox-LDL (100 mg/L) for 24 h. Cell viability (**A**), LDH activity in the media (**B**) and cell apoptosis (**C**) were measured using the MTT assay, a kit and flow cytometry, respectively. (**D**) Immunofluorescence experiments revealed the expression of P65, which was labeled by Cy3 (red), and nuclei, which were stained with DAPI (blue). Representative fluorescent images are shown. Scale bar = 20 μm. (**E**) The protein levels of Fas/FasL pathway-related molecules were analyzed by Western blot. Data are expressed as the mean ± SD of at least three independent experiments. *P < 0.05 and **P < 0.01 versus vehicle-treated control; ^#^ *P* < 0.05 and ^##^ *P* < 0.01 versus ox-LDL treatment.

We determined the protective effect of D4F on ox-LDL-induced cell injury using the MTT assay and LDH activity detection. Pre-incubation with different doses of D4F (12.5, 25 and 50 mg/L) increased cell viability and reduced LDH release in a dose-dependent manner compared with cells treated with ox-LDL alone (Fig. 3A and B). Furthermore, cell apoptosis was significantly induced by the treatment with 100 mg/L ox-LDL for 24 h compared with the control group, whereas the effect of ox-LDL was attenuated to 43.5%, 39.5% and 23.9% upon D4F pretreatment at 12.5, 25 and 50 mg/L, respectively (Fig. 3C).

Immunofluorescence staining was used to observe the relocation of P65, as visualized by Cy3 labeling (red). The red stain-positive area was located mainly in the plasma in untreated control cells. However, the positive fluorescent area was more visible within the nucleus when cells were treated with 100 mg/L ox-LDL for 24 h. Preincubation with D4F decreased the intranuclear red-stained areas compared with the ox-LDL group in a dose-dependent manner (Fig. 3D). Additionally, the results of Western blot (Fig. 3E) showed that the levels of Fas/FasL pathway-related molecules, including Fas, FasL, FADD, cleaved-caspase-8 and cleaved-caspase-3, were decreased in response to treatment with D4F in a concentration-dependent manner compared with the ox-LDL group. These data indicated that D4F may suppress the activation of the NF-κB-Fas/FasL pathway in RAW264.7 cells induced by ox-LDL.

To further confirm that the inhibitory effect of D4F on the NF-κB-Fas/FasL pathway-mediated apoptosis in macrophages induced by ox-LDL was similar to that of apoA-I, full-length apoA-I was used as a positive control. As shown in Supplementary Fig. S2, similar results were obtained in D4F- and Apo A-I-pretreated cells, as assessed by the reduced cell apoptosis, P65 nuclear translocation and the attenuated upregulation of Fas, FasL and FADD. These data indicated that similar to Apo A-I, D4F may inhibit ox-LDL-induced macrophage apoptosis by suppressing the NF-κB-Fas/FasL pathway.

### Jo2 reverses the protective effects of D4F on ox-LDL-treated RAW264.7 cells

**Figure 4:**
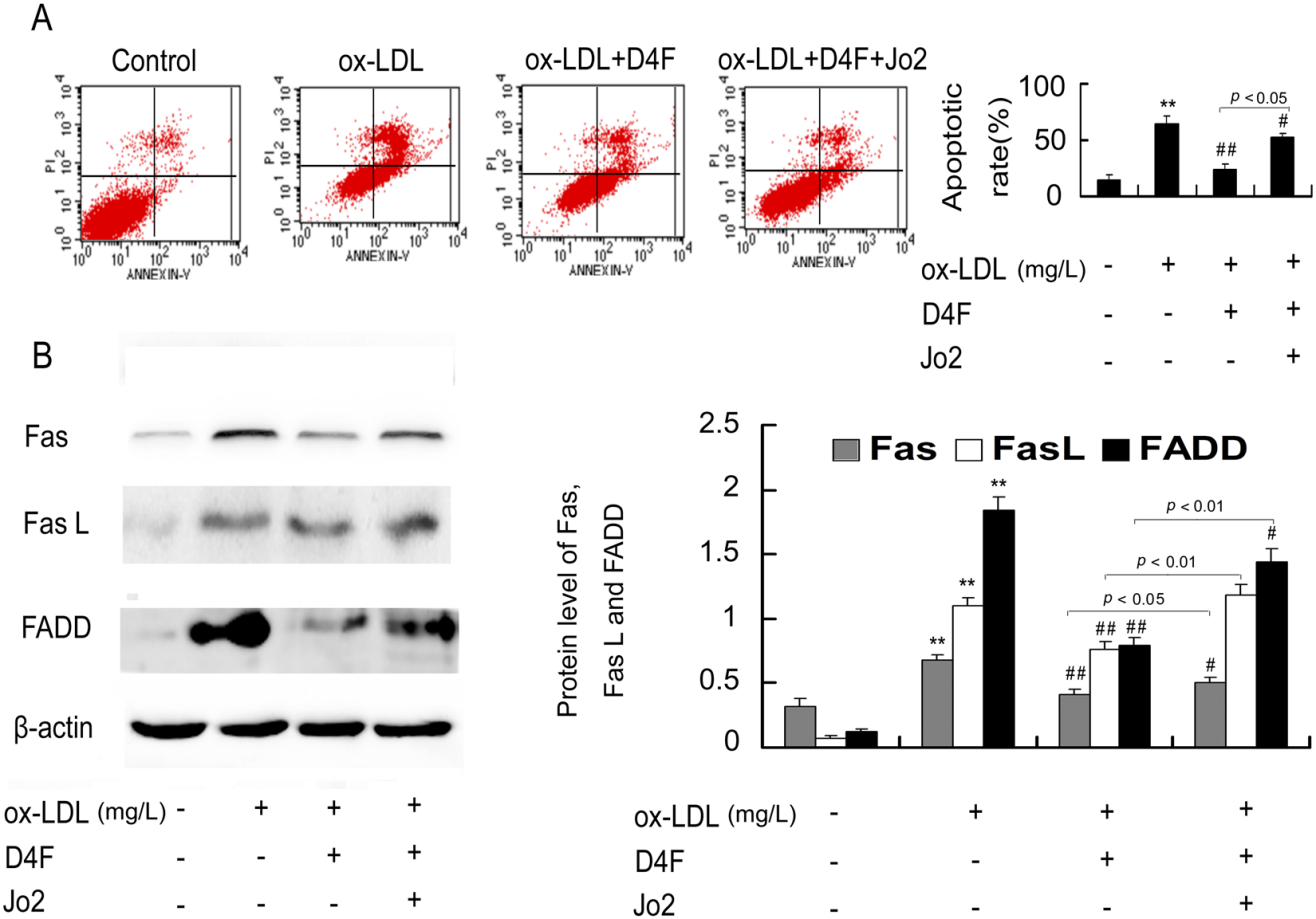
Jo2 reverses the protective effects of D4F on ox-LDL-treated RAW264.7 cells. Cells were pretreated with Jo2 (a Fas-activating antibody, 10 μg/mL) or D4F (50 mg/L) for 1 h, and then 100 mg/L ox-LDL was added to the cells for 24 h. Cell apoptosis and the protein levels of Fas, FasL and FADD were detected using flow cytometry (**A**) and Western blot (**B**), respectively. Data are expressed as the mean ± SD of at least three independent experiments. *P < 0.05 and **P < 0.01 versus vehicle-treated control; ^#^ *P* < 0.05 and ^##^ *P* < 0.01 versus ox-LDL treatment.

It has been reported that Jo2 induces Fas-mediated cell apoptosis by interacting with Fas antigen and contributing to the formation of DISC^31, 32^. To further confirm that D4F inhibits ox-LDL-induced macrophage apoptosis through attenuating the Fas/FasL pathway, we explored whether Jo2 could block the protective effect of D4F on ox-LDL-treated RAW264.7 cells. As seen in Fig. 4, the inhibitory effect of D4F on ox-LDL-induced cell apoptosis and the upregulation of Fas, FasL and FADD were obviously reversed by Jo2.

### Effects of D4F on the expression of P65 and Fas/FasL pathway-related molecules in apoE^−/−^ mice fed a high-fat diet

**Figure 5:**
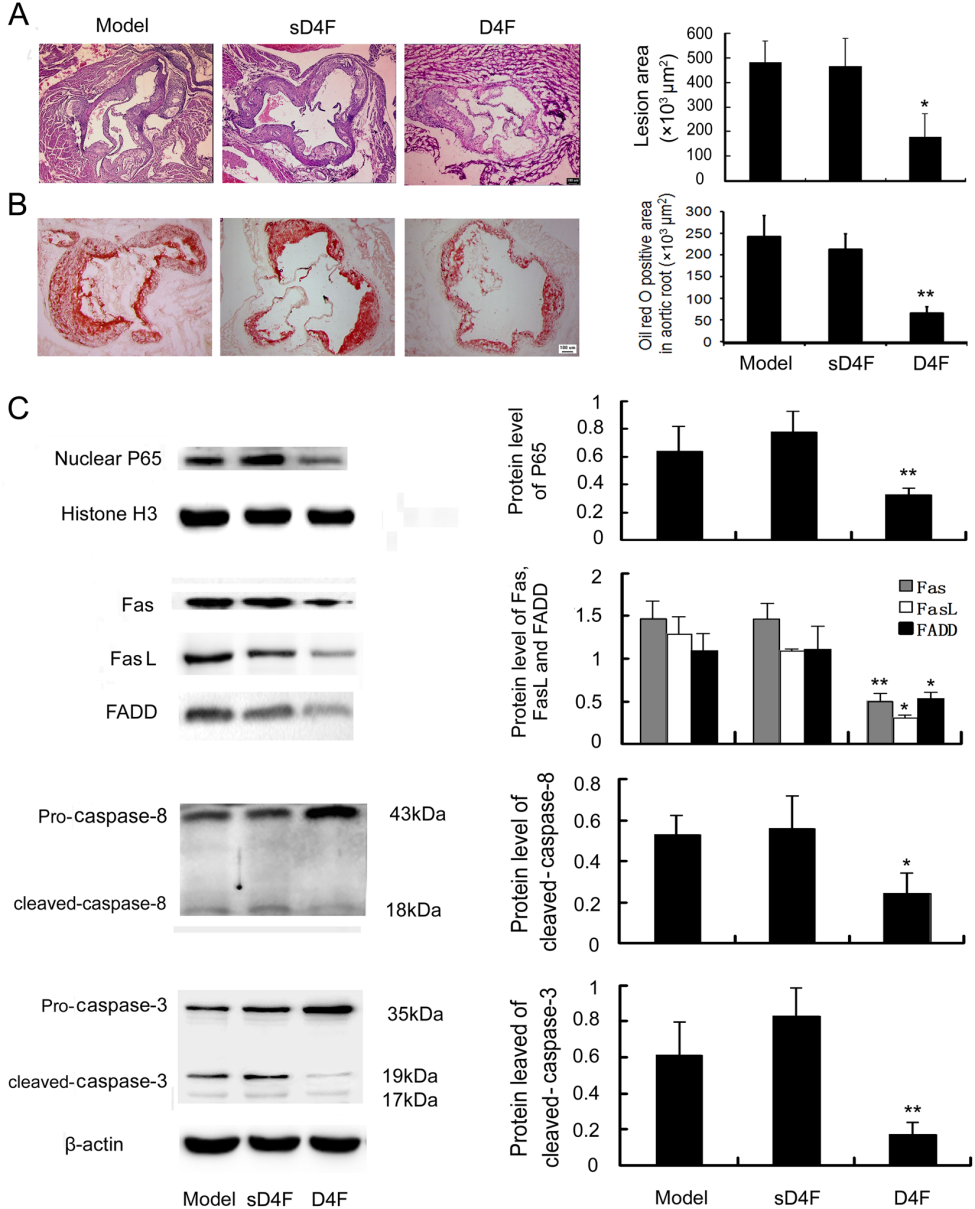
D4F reduces the atherosclerotic lesion area in aortic root and downregulates the expression of P65 and Fas/FasL pathway-related molecules in thoracic aorta. Male apoE^−/−^ mice were fed a high-fat diet for 8 weeks and given saline (model group, n = 8), 1 mg/kg sD4F (sD4F group, n = 8) or 1 mg/kg D4F (D4F group, n = 8) per day by intraperitoneal injection during the final 6 weeks. (**A**) Atherosclerotic lesion formation shown with HE staining. Scale bar = 100 μm. (**B**) Atherosclerotic lesion formation stained by oil red O. Scale bar = 100 μm. (**C**) Western blot results showing the protein levels of P65 and Fas/FasL pathway-related molecules in thoracic aorta. Data are expressed as the mean ± SD of at least three independent experiments. *P < 0.05 and **P < 0.01 versus model group.

**Figure 6:**
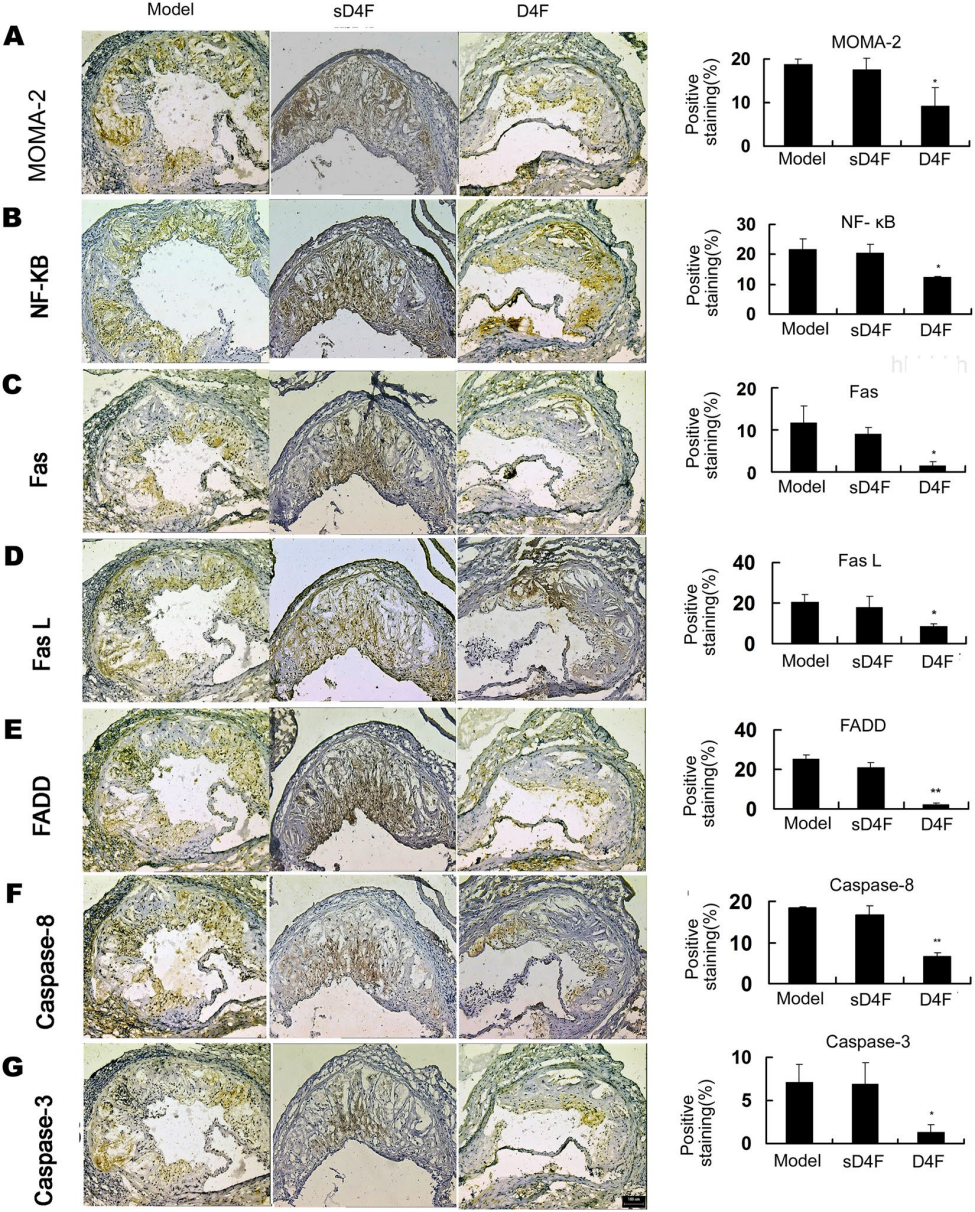
D4F inhibits the expression of P65 and Fas/FasL pathway-related molecules in atherosclerotic lesions of apoE^−/−^ mice. Male apoE^−/−^ mice were treated as described in Fig. 5. Immunohistochemical staining with specific antibodies against MOMA-2 (**A**), P65 (**B**), Fas (**C**), FasL (**D**), FADD (**E**), caspase-8 (**F**) and caspase-3 (**G**). Scale bar = 100 μm. Blue indicates nuclei and brown indicates target protein. Densitometric quantification of MOMA-2, P65, Fas, FasL, FADD, cleaved-caspase-8 and cleaved-caspase-3 per field of view was calculated. Data are presented as the mean ± SD of eight independent experiments. *P < 0.05 and **P < 0.01 versus model group.

HE staining and oil red O staining results showed that the lesion area and lipid accumulation in aortic roots of apoE^−/−^ mice were significantly decreased in the D4F-treated group compared with the model group (Fig. 5A and B), although there was no significant differences in serum cholesterol levels between the D4F and model groups (Supplementary Fig. S3). Additionally, the results of Western blot using thoracic aorta samples showed that P65 level in the nuclei and the protein expression of Fas, FasL, FADD, cleaved-caspase-8 and cleaved-caspase-3 were decreased by D4F (Fig. 5C). Similarly, immunohistochemical staining of plaque lesions in aortic roots showed that the expression of P65 and Fas/FasL pathway-related molecules in the macrophage-dense areas (shown by staining with MOMA-2) was significantly decreased by treatment with D4F (Fig. 6). These results indicate that D4F treatment may attenuate the activation of NF-κB-Fas/FasL pathway and then inhibit the development of atherosclerotic plaques.

## Discussion

Macrophage apoptosis occurs throughout all stages of atherosclerosis and plays an important role in plaque progression and instability, which may contribute to the majority of cardiovascular complications^33^. Thus, the inhibition of macrophage apoptosis may have important significance for preventing the progression of AS and reducing the incidence of cardiovascular disease events. In this study, our results indicate for the first time that NF-κB mediates Fas/FasL pathway activation and apoptosis in macrophages induced by ox-LDL and that D4F alleviates ox-LDL-induced macrophage apoptosis by inhibiting the activation of the NF-κB-Fas/FasL pathway, which is supported by the following observations. First, ox-LDL induces apoptosis by activating the Fas/FasL death receptor apoptotic pathway in RAW264.7 cells, which is regulated by NF-κB. Second, D4F inhibits ox-LDL-induced macrophage apoptosis, P65 nuclear translocation and the upregulation of Fas/FasL pathway-related molecules, which are blocked by Jo2 (a Fas-activating monoclonal antibody). Third, D4F inhibits the expression of P65, Fas, FasL and FADD as well as downstream cleaved-caspase-8/3 in atherosclerotic plaques of apoE^−/−^ mice fed a high-fat diet.

Fas is found to be co-localized with CD68-positive macrophage-derived foam cells in and around the areas of plaque that exhibit features of a necrotic core in human aortic atheromatous lesion samples^34^. Adenoviral transfection of Fas can reduce the number of cells in the fibrous cap and increase thrombus formation and bleed inside the plaque, thereby promoting plaque vulnerability^13^. Ox-LDL dramatically increases the levels of Fas/FasL pathway-related molecules, whereas pretreatment with ZB4 antibody (an antagonist antibody that disturbs Fas/FasL interaction) obviously blocks ox-LDL-induced apoptosis in macrophages14. In the present work, we showed that ox-LDL induced apoptosis and upregulated the expression of Fas/FasL pathway-related proteins in RAW264.7 cells, whereas the silencing of Fas blocked ox-LDL-induced macrophage apoptosis. These data suggest that activation of the Fas/FasL pathway may be an important mechanism underlying ox-LDL-induced macrophage apoptosis. NF-κB has an important role in autoimmune and inflammatory responses, oxidative stress and apoptosis regulation, and it is an important therapeutic target for AS^35, 36^. Accumulating data have shown that NF-κB can be directly integrated into the Fas and FasL promoter and increase the expression of Fas and FasL in tumor cells to induce cell apoptosis^37–39^. It has been reported that NF-κB activation is accompanied by the overexpression of Fas, FasL and cleaved-caspase-3 in apoptotic cells within carotid artery plaques of patients^12^. Our results in the present study showed that P65, Fas, FasL, FADD, cleaved-caspase-8 and cleaved-caspase-3 partially overlapped with MOMA2-positive macrophages in serial atherosclerotic sections from apoE^−/−^ mice fed a high-fat diet. In addition, the silencing of P65 attenuated macrophage apoptosis and the upregulation of Fas caused by ox-LDL, whereas P65 expression was not significantly affected by treatment with Fas siRNA. These results indicate that NF-κB may mediate the activation of the Fas pathway and then cause macrophage apoptosis. D4F, an apoA-I mimetic peptide, has been demonstrated to exert a variety of atheroprotective mechanisms, such as inhibiting oxidative stress and inflammation, improving cholesterol efflux from foam cells, regulating the level of plasma cholesterol and increasing the formation of pre-beta1 HDL in human plasma^17–19, 21, 23, 40, 41^. Additionally, chronic D4F treatment significantly decreases circulating endothelial cell injury and superoxide anion production, and improves the impaired vascular reactivity in diabetic rats^42^. Furthermore, D4F enhances isoflurane-induced eNOS signaling and cardioprotection during acute hyperglycemia^43^, and promotes proliferation, migration and adhesion of circulating endothelial progenitor cells as well as the survival of vascular endothelial cells via the endothelial nitric oxide (NO) synthase/NO pathway^25^. In this study, we observed that D4F attenuated apoptosis, P65 nuclear translocation and the upregulation of Fas, FasL, FADD as well as downstream effector caspases (caspase-8 and caspase-3) in RAW264.7 cells induced by ox-LDL and in atherosclerotic lesions of apoE^−/−^ mice. However, Jo2, a Fas-activating monoclonal antibody, obviously reversed the inhibitory effect of D4F on ox-LDL-induced cell apoptosis and the upregulation of Fas, FasL and FADD. These results indicate that D4F may protect macrophages from ox-LDL-induced apoptosis by inhibiting the activation of NF-κB and the Fas/FasL pathway, consequently attenuating atherosclerotic development.

In conclusion, the present study indicates that NF-κB mediates Fas/FasL pathway activation and apoptosis in macrophages induced by ox-LDL and that D4F protects macrophages from ox-LDL-induced apoptosis through inhibiting the NF-κB- dependent Fas/FasL pathway. The results might further explain the diverse physiological activities of D4F and support the pharmacological application of D4F for atherosclerosis-related diseases.

## Materials and Methods

### Materials

D4F (Ac-DWFKAFYDKVAEKFKEAF-NH_2_) and scrambled D4F (Ac-DWFAK DYFKKAFVEEFAK-NH_2_) were synthesized by Scilight Biotechnology (Beijing, China). Ox-LDL was from Xiesheng Biotech (Beijing, China). Anti-MOMA2, anti-Fas and anti-Fas ligand (FasL) antibodies were purchased from Abcam (Cambridge, MA, USA). Antibodies against nuclear factor-κB (NF-κB) P65, Histone H3, caspase-8 and caspase 3 were purchased from Santa Cruz Biotech (Santa Cruz, CA, USA). Anti-FADD antibody were purchased from Abclonal technology (Cambridge, MA, USA). Horseradish peroxidase-labeled goat anti-rabbit IgG and goat anti-rat IgG as well as the rabbit or rat primary antibody immunohistochemistry kits were obtained from ZSGB-BIO (Beijing, China). RIPA lysis buffer and bicinchoninic acid (BCA) protein quantitation kits were purchased from Solarbio (Beijing, China). Enhanced chemiluminescence (ECL) kits and polyvinylidene fluoride (PVDF) membranes were purchased from Thermo Scientific Pierce (Rockford, IL, USA) and Millipore (Bedford, USA), respectively. Dulbecco’s modified Eagle’s medium (DMEM) and fetal bovine serum (FBS) were purchased from Gibco (BRL, Gaithersburg, MD, USA). Jo2 (Purified NA/LE Hamster Anti-Mouse Fas), Annexin V-FITC and propidium iodide (PI) were obtained from BD Biosciences (San Jose, CA, USA). Anti-β-actin antibody, the short interfering RNA (siRNA) against Fas and apo A-I were obtained from Sigma-Aldrich (St Louis, MO, USA). 3-(4,5-dimethylthiazol-2-y-l)-2,5-diphenyl-2H-tetrazolium bromide (MTT) and lactate dehydrogenase (LDH) assay kits were from Genview (Houston, TX, USA) and Jiancheng Biotech (Nanjing, China), respectively. NF-κB P65 translocation kits were purchased from Beyotime Biotech (Shanghai, China).

### Cell culture

RAW264.7 cells, a murine macrophage cell line (Type Culture Collection of the Chinese Academy of Sciences, Shanghai, China), were cultured in DMEM containing 10% FBS, 2 mmol/L glutamine and 1% antibiotics (penicillin A and streptomycin), and maintained at 37 °C in a humidified atmosphere containing 5% CO_2_. The medium was replaced with serum-free medium for 12 h before treatment.

### Cell viability and LDH assays

Following treatment, the viability of RAW264.7 cells grown in 96-well plates was evaluated using the MTT assay as previously described^44^. Optical intensity (OD) was assessed with an Infinite F200 microplate reader (Tecan) at a wavelength of 490 nm, and viability was expressed as a percentage of the control.

The release of cytosolic LDH into the medium was used as a generic index of cell injury. After treatment, the media were collected and assayed for LDH activity using the assay kit based on the manufacturer’s instructions.

### Flow cytometry analysis of apoptotic cells

The Annexin V-FITC/PI double-staining assay was used to quantify apoptosis following the manufacturer’s protocol. After treatment, the cells were harvested, washed twice with ice-cold PBS and centrifuged. A 500-μl volume of binding buffer was added to resuspend the cells, and then, 10 μl of Annexin V- FITC and 5 μl of PI were added. The cells were incubated for 15 min in the dark, and the apoptotic cells were detected within 30 min. The samples were analyzed on a FACScan flow cytometer using CellQuest software (Becton Dickinson, San Jose, CA, USA).

### SiRNA Transfection

RAW264.7 cells were transfected with specific siRNA oligomers directed against P65 and Fas (80 nM) using Lipofectamine 2000 transfection reagent (Invitrogen, Carlsbad, CA, USA) according to the manufacturer’s instructions, as previously described^45^.

### P65 nuclear translocation assay

After the experimental procedures, the cells grown on glass coverslips were washed with PBS, fixed with 4% paraformaldehyde and permeabilized with 0.1% Triton. Next, cells were blocked with 3% BSA and incubated for 1 h with P65 antibody (1/200). The slides were then washed and incubated with Cy3-labeled anti-rabbit IgG (1/500) for 1 h, and the nucleus was stained with DAPI for 5 min. Stained cells were observed using an Olympus BX51 microscope (Tokyo, Japan) as previously described^44^.

### Animal Protocol

Male apolipoprotein E knockout (ApoE^−/−^) mice (7 weeks of age) were obtained from the Huafukang Bio-Technology Company (Beijing, China). A total of 24 ApoE^−/−^ mice fed a high-fat diet (15.8% fat and 1.25% cholesterol) for 8 weeks were randomly intraperitoneally injected with saline (model group, n = 8), sD4F (1 mg/kg per day, sD4F group, n = 8) or D4F (1 mg/kg per day, D4F group, n = 8) during the final 6 weeks. At the end of the experiment, the mice were euthanized by cervical dislocation, and the hearts were perfused with ice-cold saline. They were subsequently removed transversely, and then the hearts, including the aortic root, were fixed in 4% paraformaldehyde and paraffin embedded. All experiments were approved by the laboratory animals’ ethics committee of Taishan Medical University and followed the national guidelines for the care and use of animals.

### Histology and immunohistochemistry

To assess the atherosclerotic lesions and lipid accumulation, serial aortic root sections were stained with hematoxylin/eosin (HE) and oil red O. Atherosclerotic lesions were captured as digital images using a microscope (Olympus, Tokyo, Japan). The total mean lesion area and the mean oil red O positive area were quantified from five sections per animal using Image-Pro Plus software (version 6.0; Media Cybernetics, MD, USA). Immunohistochemical staining with specific antibodies against macrophages (MOMA-2), P65, Fas, FasL, FADD, caspase-8 and caspase-3 was performed following the manufacturer’s recommendations. The sections were overlaid with biotinylated secondary antibodies, visualized with a streptavidin- peroxidase/diaminobenzidine system and counterstained with hematoxylin. Color threshold and planimetry were used for quantitation. All quantitations were determined by calculating the percentage of antigen-positive area compared to the total cross-sectional vessel wall area using Image-Pro Plus software.

### Western blot analysis

Total protein and nuclear protein from the tissues or cells were extracted as previously described^45^. They were subjected to Western blot analysis using anti-P65, anti-Fas, anti-FasL, anti-FADD, anti-caspase-8 and anti-caspase-3 antibodies. The proteins were visualized using an ECL method, and then, the integrated optical density (IOD) of the immunoreactive bands was measured using Image-Pro Plus software and normalized to β-actin or Histone H3 levels.

### Statistical analysis

Results were expressed as the mean ± SD. Statistical analysis was performed by one-way analysis of variance with the Student–Newmann–Keuls test using SPSS13.0 software for Windows. Statistical differences were considered significant at a *P* value less than 0.05.

## Electronic supplementary material

Supplementary Figures R1
