# Supplementary material for: D4F alleviates macrophage-derived foam cell apoptosis by inhibiting the NF-κB-dependent Fas/FasL pathway
Source: Sci Rep. 2017 Aug 4;7:7333. doi: 10.1038/s41598-017-07656-0 (PMC5544683; doi:10.1038/s41598-017-07656-0)
Supplement: Supplementary file 1 — Supplementary Figures R1 [file 41598_2017_7656_MOESM1_ESM.pdf]

D4F alleviates macrophage-derived foam cell apoptosis by inhibiting the NF-κB-dependent Fas/FasL pathway

Hua Tian[1], Shu-tong Yao[1, 2*], Na-na Yang[1], Jie Ren[3], Peng Jiao[1], Xiangjian Zhang[4], Dong-xuan Li[1], Gong-an Zhang[1], Zhen-fang Xia[1], Shu-cun Qin[1*]

[1]Key Laboratory of Atherosclerosis in Universities of Shandong, Institute of Atherosclerosis, Taishan Medical University, Taian 271000, China

[2] College of Basic Medical Sciences, Taishan Medical University, Taian 271000, China

[3]Institute of Cardiovascular Disease, General Hospital of Jinan Military Region, Jinan, 250022, China

[4]Hebei Collaborative Innovation Center for Cardio-cerebrovascular Disease and Hebei Key Laboratory of Vascular Homeostasis, Shijiazhuang 050000, China

* Corresponding author at: Institute of Atherosclerosis, Taishan Medical University, 2# Yingsheng East Road, Taian 271000, Shandong, China. Tel: +86-538-6237252; Fax: +86-538-6225275: *E-mail addresses*: yst228@126.com (S. Yao), shucunqin@ hotmail.com (S. Qin).

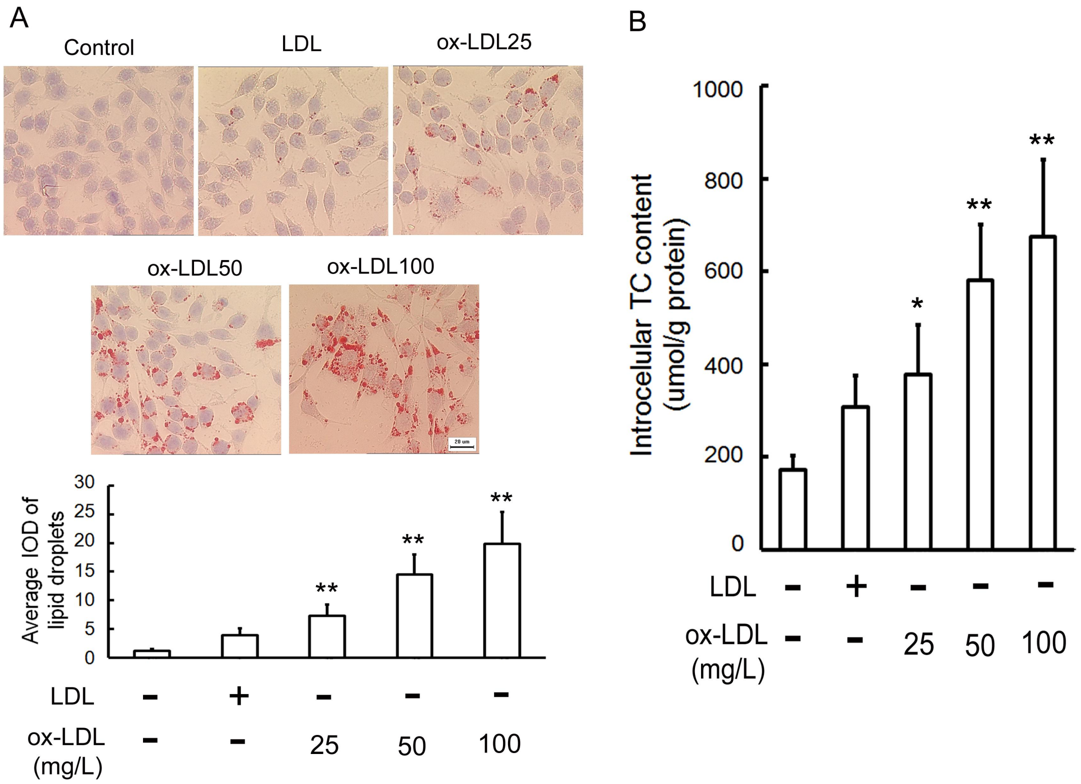

Supplementary Fig. S1. Ox-LDL induces intracellular lipid accumulation in RAW264.7 cells. Cells were treated with ox-LDL (25, 50 and 100 mg/L) or LDL (100 mg/L) for 24 h. (A) The intracellular lipid droplets were stained by oil red O. Representative images of lipid droplet staining are shown. Scale bar=20 μm. The average integrated optical density (IOD) of lipid droplets stained with oil red O in differentiated macrophage-derived foam cells was obtained by checking five fields in each group. (B) The intracellular total cholesterol (TC) content was detected using a tissue/cell TC assay kit. Data are expressed as the mean ± SD of at least four independent experiments. *$P < 0.05$ and **$P < 0.01$ versus vehicle-treated control.

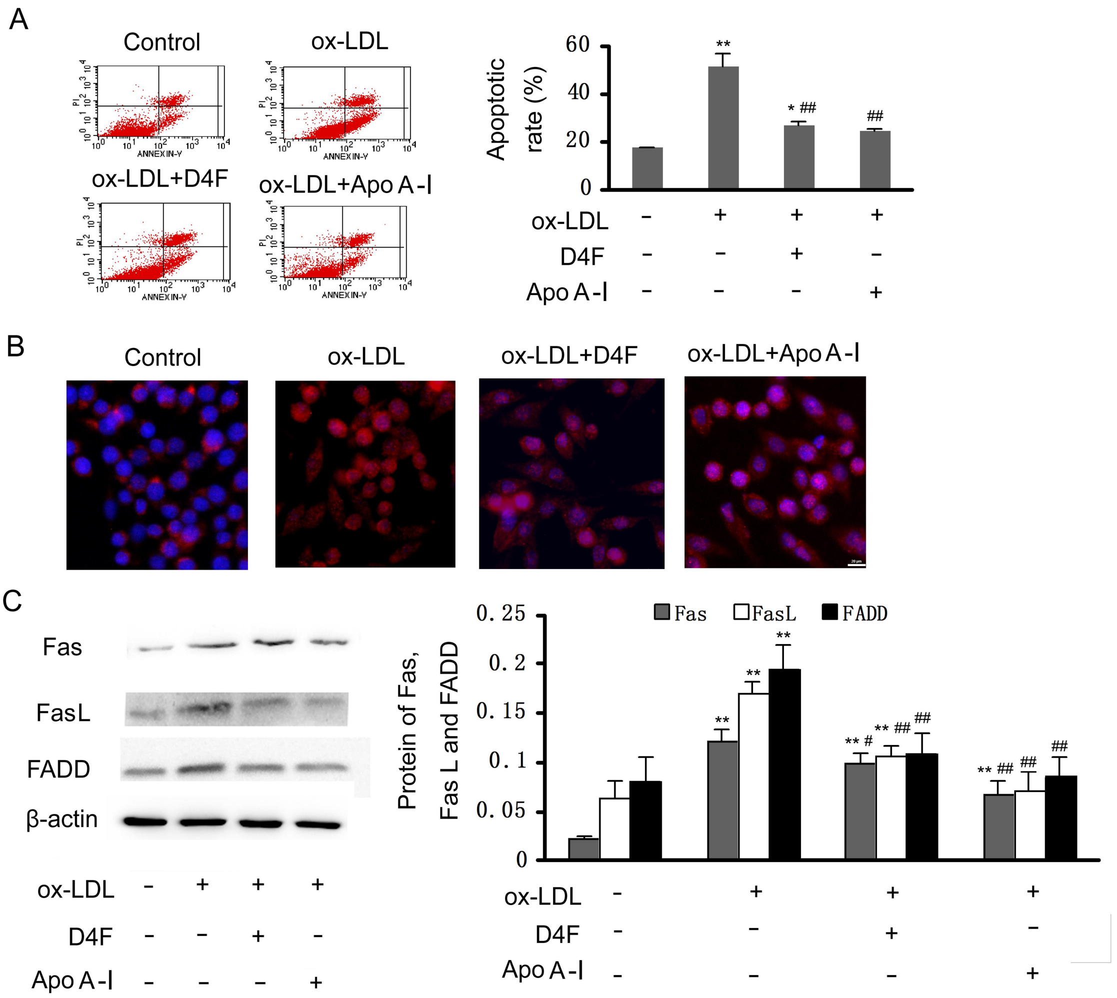

Supplementary Fig. S2. Effects of D4F and Apo A-I on apoptosis, P65 nuclear translocation and the activation of Fas/FasL pathway in RAW264.7 cells induced by ox-LDL. RAW264.7 cells were pretreated with D4F (50 mg/L) or Apo A-I (50 mg/L) for 1 h followed by incubation with ox-LDL (100 mg/L) for 24 h. Cell apoptosis (A) was measured using flow cytometry, and immunofluorescence experiments showed P65 expression using Cy3 labeling (red), and the nuclei were stained with DAPI (blue). Representative fluorescent images are shown (B). Scale bar=20 μm. The protein levels of Fas, FasL and FADD were analyzed by Western blot (C). Data are expressed as the mean ± SD of at least three independent experiments. **P* < 0.05 and ***P* < 0.01 versus vehicle-treated control; #*P* < 0.05 and ##*P* < 0.01 versus ox-LDL treatment.

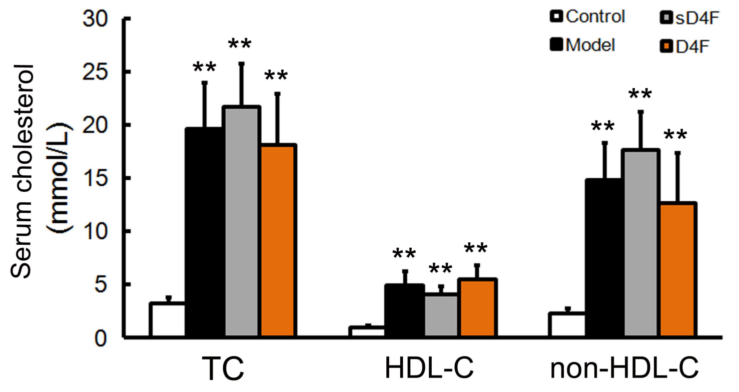

Supplementary Fig. S3. Effects of D4F on serum cholesterol levels in apoE$^{-/-}$ mice. Male apoE$^{-/-}$ mice were fed a high-fat diet for 8 weeks and given saline (model group, n=8), 1 mg/kg sD4F (sD4F group, n=8) or 1 mg/kg D4F (D4F group, n=8) per day by intraperitoneal injection during the final 6 weeks. Male C57BL/6J mice were maintained on normal chow diet as a control group. Levels of serum total cholesterol (TC) and high density lipoprotein-cholesterol (HDL-C) in mice at the end of experiment were determined by commercial assay kits. Non-HDL-C was calculated as TC minus HDL-C. Data are presented as the mean ± SD of eight independent experiments. ***P*<0.01 versus control group.
